# Supplementary material for: Flower-mediated plant-butterfly interactions in an heterogeneous tropical coastal ecosystem
Source: PeerJ. 2018 Sep 7;6:e5493. doi: 10.7717/peerj.5493 (PMC6130237; doi:10.7717/peerj.5493)
Supplement: Table S1 — CDS, coastal dune scrub; PDV, pioneer dune vegetation; TDF, tropical deciduous forest; TDF-W, tropical deciduous flooding forest and wetland; TSF, tropical sub-deciduous forest. The nomenclature is based on official web pages (https://www.butterfliesandmoths.org/; http://janzen.sas.upenn.edu/; http://www.butterfliesofamerica.com/). [file peerj-06-5493-s001.docx]

**Supplementary material 1.** List of butterfly species per vegetation type and common plant species on which they feed. CDS = coastal dune scrub, PDV = pioneer dune vegetation, TDF = tropical deciduous forest, TDF-W = tropical deciduous flooding forest and wetland, TSF = tropical sub-deciduous forest. The nomenclature is based on official web pages (https://www.butterfliesandmoths.org/; http://janzen.sas.upenn.edu/; http://www.butterfliesofamerica.com/).

| **Family** | **Butterfly species** | **CDS** | **PDV** | **TDF** | **TDF-W** | **TSF** | **Common feeding plant species** |
| --- | --- | --- | --- | --- | --- | --- | --- |
| **Crambidae** | *Pyrausta tyralis* Guenée, 1854 | **X** | **X** |  |  |  | *Bidens pilosa* L.; *Palafoxia lindenii* A.Gray |
| **Erebidae** | *Horama oedippus* (Boisduval, 1870) | **X** | **X** | **X** | **X** | **X** | *Ageratum corymbosum* Zuccagni ex Pers. |
|  | *Horama plumipes* (Drury, 1773) |  | **X** |  |  |  | *Ageratum corymbosum* Zuccagni ex Pers.; *Caesalpinia bonduc* (L.) Roxb. |
|  | *Horama* sp. |  | **X** |  |  |  | *Ageratum corymbosum* Zuccagni ex Pers. |
|  | *Macrocneme* sp. |  | **X** |  |  |  | *Metastelma schlechtendalii* (Decne.) Standl. & Steyerm. |
|  | *Utetheisa ornatrix* (Linnaeus, 1758) |  | **X** |  |  |  | *Crotalaria incana* L. |
| **Geometridae** | *Melanchroia chephise* (Stoll, 1782) | **X** | **X** |  |  |  | *Bidens pilosa* L. |
| **Hesperiidae** | Sp. 1 (Hesp043) | **X** |  |  | **X** |  | *Pontederia sagittata* C. Presl |
|  | Sp. 2 (Hesp044) |  | **X** |  | **X** |  | *Bidens pilosa* L. |
|  | Sp. 3 (Hesp048) |  |  |  | **X** |  | *Thalia geniculata* L. |
|  | Sp. 4 (Hesp085) |  | **X** |  |  |  | *Palafoxia lindenii* A.Gray |
|  | Sp. 5 (Hesp087) |  | **X** |  |  |  | *Porophyllum punctatum* (Mill.) S.F.Blake |
|  | Sp. 6 (Hesp159) |  |  | **X** |  |  | *Lantana camara* L. |
|  | Sp. 7 (Hesp162) | **X** | **X** |  |  |  | *Cnidoscolus herbaceus* (L.) I.M. Johnst. |
|  | Sp. 8 (Hesp163) |  | **X** |  |  |  | *Ipomoea pes-caprae* (L.) Sweet |
|  | Sp. 9 (Hesp168) |  |  |  | **X** |  | *Sagittaria lancifolia* L. (M) |
|  | *Antigonus erosus* (Hübner, [1812]) |  | **X** |  |  |  | *Bidens pilosa* L.; *Caesalpinia bonduc* (L.) Roxb. |
|  | *Antigonus* *nearchus* (Latreille, 1817) |  | **X** |  |  |  | *Caesalpinia bonduc* (L.) Roxb. |
|  | *Astraptes fulgerator azul* (Reakirt, [1867]) |  |  |  | **X** |  | *Carica papaya* L. |
|  | *Calpodes ethlius* (Stoll, 1782) |  | **X** |  | **X** |  | *Thalia geniculata* L. |
|  | *Chioides albofasciatus* (Hewitson, 1867) | **X** | **X** |  | **X** |  | *Thalia geniculata* L. |
|  | *Cogia* *calchas* (Herrich-Schäffer, 1869) | **X** | **X** | **X** |  |  | *Waltheria indica* L. |
|  | *Corticea* *lysias* *lysias* (Plötz, 1883) |  | **X** |  | **X** |  | *Ipomoea pes-caprae* (L.) Sweet |
|  | *Cycloglypha thrasibulus thrasibulus* (Fabricius, 1793) | **X** | **X** | **X** |  |  | *Bidens pilosa* L. |
|  | *Cyclosemia* *anastomosis* Mabille, 1878 |  |  |  | **X** |  | *Thalia geniculata* L. |
|  | *Epargyreus spina spina* Evans, 1952 | **X** | **X** |  |  |  | *Caesalpinia bonduc* (L.) Roxb. |
|  | *Heliopetes arsalte* (Linnaeus, 1758) |  | **X** |  | **X** |  | *Phyla nodiflora* (L.) Greene |
|  | *Heliopetes macaira macaira* (Reakirt, [1867]) | **X** |  |  |  |  | *Florestina pedata* (Cav.) Cass. |
|  | *Heliopyrgus sublinea* (Schaus, 1902) |  | **X** | **X** |  |  | *Tamonea curassavica* (L.) Pers. |
|  | *Hylephila phyleus phyleus* (Drury, 1773) |  | **X** |  | **X** |  | *Bidens pilosa* L. |
|  | *Lerema* *liris* Evans, 1985 |  | **X** |  |  |  | *Ipomoea pes-caprae* (L.) Sweet |
|  | *Lerodea* *eufala* *eufala* (W. H. Edwards, 1869) |  |  | **X** |  |  | *Tamonea curassavica* (L.) Pers. |
|  | *Mylon pelopidas* (Fabricius, 1793) | **X** | **X** |  | **X** |  | *Caesalpinia bonduc* (L.) Roxb. |
|  | *Nisoniades* *godma* Evans, 1953 |  | **X** |  |  |  | *Ageratum corymbosum* Zuccagni ex Pers. |
|  | *Nisoniades macarius* (Herrich-Schäffer, 1870) | **X** | **X** |  | **X** | **X** | *Sagittaria lancifolia* L. (M) |
|  | *Panoquina hecebolus* (Scudder, 1872) | **X** | **X** | **X** |  |  | *Caesalpinia bonduc* (L.) Roxb. |
|  | *Panoquina panoquinoides panoquinoides* (Skinner, 1891) |  | **X** |  |  | **X** | *Bidens pilosa* L. |
|  | *Phocides belus* Godman & Salvin, 1890 |  | **X** |  |  |  | *Palafoxia lindenii* A.Gray |
|  | *Polites vibex vibex* (Geyer, 1832) |  | **X** |  |  |  | *Bidens pilosa* L. |
|  | *Polythrix asine* (Hewitson, 1867) |  | **X** | **X** |  |  | *Caesalpinia bonduc* (L.) Roxb. |
|  | *Proteides mercurius mercurius* (Fabricius, 1787) |  | **X** |  |  |  | *Caesalpinia bonduc* (L.) Roxb. |
|  | *Pyrgus communis communis* (Grote, 1872) |  | **X** |  |  |  | *Bidens pilosa* L. |
|  | *Quasimellana eulogius* (Plötz, 1882) |  |  | **X** |  |  | *Lysiloma divaricatum* (Jacq.) J.F. Macbr. |
|  | *Staphylus* *azteca* (Scudder, 1872) |  |  | **X** |  |  | *Turnera diffusa* Willd. |
|  | *Staphylus mazans* (Reakirt, [1867]) | **X** | **X** | **X** |  |  | *Waltheria indica* L. |
|  | *Thessia jalapus* (Plötz, 1881) | **X** | **X** |  | **X** |  | *Thalia geniculata* L. |
|  | *Urbanus dorantes dorantes* (Stoll, 1790) | **X** | **X** |  | **X** | **X** | *Petrea volubilis* L. |
|  | *Urbanus doryssus* (Swainson, 1831) (E Mexican segregate) |  |  |  | **X** |  | *Calopogonium caeruleum* (Benth.) C. Wright |
|  | *Urbanus esmeraldus* (Butler, 1877) | **X** |  |  | **X** |  | *Calopogonium caeruleum* (Benth.) C. Wright |
|  | *Urbanus procne* (Plötz, 1881) |  |  |  | **X** |  | *Pontederia sagittata* C. Presl |
|  | *Urbanus proteus proteus* (Linnaeus, 1758) | **X** | **X** | **X** | **X** |  | *Thalia geniculata* L.; *Sagittaria lancifolia* L. (M); C*hrysobalanus icaco* L. |
|  | *Urbanus simplicius* (Stoll, 1790) | **X** | **X** |  | **X** |  | *Macroptilium atropurpureum* (Moc. & Sessé ex DC.) Urb. |
|  | *Virga* *clenchi* L. Miller, 1970 |  |  |  | **X** |  | *Sagittaria lancifolia* L. (M) |
| **Lycaenidae** | *Arawacus sito* (Boisduval, 1836) |  | **X** |  | **X** |  | *Bidens pilosa* L.; *Sagittaria lancifolia* L. (M) |
|  | *Calycopis isobeon* (Butler & H. Druce, 1872) | **X** | **X** |  | **X** |  | *Bidens pilosa* L.; *Cordia spinescens* L. |
|  | *Hemiargus ceraunus astenidas* (Lucas, 1857) | **X** | **X** |  |  |  | *Bidens pilosa* L. |
|  | *Hemiargus ceraunus watsoni* W. Comstock & Huntington, 1943 | **X** | **X** | **X** |  |  | *Bidens pilosa* L. |
|  | *Leptotes cassius cassidula* (Boisduval, 1970) | **X** | **X** | **X** | **X** |  | *Bidens pilosa* L. |
|  | *Michaelus hecate* (Godman & Salvin, 1887) | **X** |  | **X** |  |  | *Chrysobalanus icaco* L. |
|  | *Ministrymon inoa* (Godman & Salvin, 1887) |  |  | **X** |  |  | *Turnera diffusa* Willd. |
|  | *Panthiades bathildis* (C. Felder & R. Felder, 1865) |  | **X** | **X** | **X** |  | *Macroptilium atropurpureum* (Moc. & Sessé ex DC.) Urb. |
|  | *Panthiades bitias* (Cramer, 1777) |  |  |  | **X** |  | *Cordia spinescens* L. |
|  | *Rekoa palegon* (Cramer, 1780) | **X** | **X** |  |  |  | *Bidens pilosa* L. |
|  | *Strymon bazochii bazochii* (Godart, [1824]) |  |  | **X** |  |  | *Florestina pedata* (Cav.) Cass. |
|  | *Strymon istapa istapa* (Reakirt, [1867]) |  | **X** | **X** |  |  | *Bidens pilosa* L. |
|  | *Strymon serapio* (Goldman & Salvin, 1887) |  | **X** | **X** |  |  | *Chrysobalanus icaco* L. |
| **Microlepidoptera** | Moth No. 12 | **X** | **X** | **X** |  |  | *Palafoxia lindenii* A.Gray |
| **Nymphalidae** | *Adelpha basiloides* (H. Bates, 1865) |  | **X** |  | **X** |  | *Cordia spinescens* L. |
|  | *Agraulis vanillae incarnata* (N. Riley, 1926) | **X** | **X** | **X** |  |  | *Cnidoscolus herbaceus* (L.) I.M. Johnst. |
|  | *Anartia fatima fatima* (Fabricius, 1793) |  | **X** |  | **X** |  | *Asclepias curassavica* L. |
|  | *Anartia jatrophae* luteipicta Frustorfer, 1907 |  | **X** |  | **X** |  | *Bidens pilosa* L. |
|  | *Anthanassa tulcis* (H.W Bates, 1864) |  | **X** |  | **X** |  | *Bidens pilosa* L. |
|  | *Archaeoprepona demophon centralis* (Fruhstorfer, 1905) |  |  |  | **X** |  | *Tabernaemontana alba* Mill. |
|  | *Chlosyne theona theona* (Ménétriés, 1855) | **X** | **X** | **X** |  |  | *Bidens pilosa* L. |
|  | *Consul fabius cecrops* (Doubleday, [1849]) |  |  |  |  | **X** | *Jacquinia macrocarpa* Cav. |
|  | *Danaus gilippus thersippus* (H.W. Bates, 1863) | **X** | **X** | **X** | **X** |  | *Ageratum corymbosum* Zuccagni ex Pers. |
|  | *Danaus plexippus plexippus* (Linnaeus, 1785) |  | **X** |  | **X** |  | *Ageratum corymbosum* Zuccagni ex Pers. |
|  | *Dione juno huascuma* (Reakirt, 1866) | **X** |  |  |  |  | *Lantana camara* L. |
|  | *Doxocopa pavon theodora* (Lucas, 1857) | **X** |  |  |  |  | *Turnera ulmifolia* L. |
|  | *Dryas iulia moderata* (Riley, 1926) | **X** | **X** | **X** | **X** |  | *Lantana camara* L. |
|  | *Eueides aliphera gracilis* (Stichel, 1903) | **X** |  |  |  |  | *Bidens pilosa* L. |
|  | *Eunica monima* (Stoll, 1782) |  |  | **X** |  |  | *Lysiloma divaricatum* (Jacq.) J.F. Macbr. |
|  | *Euptoieta hegesia meridiania* Stichel, 1938 | **X** | **X** | **X** |  |  | *Turnera diffusa* Willd. |
|  | *Hamadryas guatemalena marmarice* (Fruhstorfer, 1916) |  |  |  | **X** |  | *Pontederia sagittata* C. Presl |
|  | *Heliconius charitonia vazquezae* W. Comstock & F. Brown, 1950 | **X** |  | **X** |  | **X** | *Lantana camara* L. |
|  | *Heliconius erato petiverana* Doubleday, 1847 | **X** |  |  | **X** | **X** | *Lantana camara* L. |
|  | *Junonia evarete* (Cramer, 1779) (E Mexican segregate) | **X** | **X** |  |  |  | *Bidens pilosa* L. |
|  | *Libytheana carinenta mexicana* Michener, 1943 | **X** |  |  |  |  | *Cordia polycephala* (Lam.) I.M.Johnst. |
|  | *Lycorea halia atergatis* Doubleday, [1847] |  |  |  | **X** |  | *Mikania micrantha* Kunth |
|  | *Marpesia chiron* (Fabricius, 1775) |  | **X** |  | **X** |  | *Cordia spinescens* L. |
|  | *Marpesia petreus* (Cramer, 1176) (Northern segregate) | **X** | **X** | **X** | **X** |  | *Cordia spinescens* L. |
|  | *Mechanitis polymia lycidice* H. Bates, 1864 |  |  |  | **X** |  | *Cordia spinescens* L. |
|  | *Myscelia ethusa ethusa* (Doyère, [1840]) |  |  |  | **X** |  | *Sagittaria lancifolia* L. (M) |
|  | *Siproeta stelenes* (Linnaeus, 1758) | **X** | **X** |  | **X** |  | *Cordia spinescens* L. |
|  | *Taygetis thamyra* (Cramer, 1779 |  |  |  | **X** |  | *Sagittaria lancifolia* L. (M) |
| **Papilionidae** | *Battus philenor philenor* (Linnaeus, 1771) |  | **X** | **X** |  |  | *Russelia sarmentosa* Jacq. |
|  | *Eurytides epidaus epidaus* (Doubleday, 1846) | **X** |  | **X** |  |  | *Lysiloma divaricatum* (Jacq.) J.F. Macbr.; *Randia* *aculeata* var. *dasyclada* Steyerm. |
|  | *Eurytides philoaus philoaus* (Boisduval, 1836) |  |  | **X** |  |  | *Lysiloma divaricatum* (Jacq.) J.F. Macbr. |
|  | *Mimoides phaon phaon* (Boisduval, 1836) | **X** |  | **X** |  |  | *Lantana camara* L. |
|  | *Papilio anchisiades idaeus* Fabricius, 1793 |  |  | **X** |  |  | *Lysiloma divaricatum* (Jacq.) J.F. Macbr. |
|  | *Papilio thoas autocles* Rostchild y Jordan, 1906 | **X** |  | **X** |  |  | *Lantana camara* L.; *Lysiloma divaricatum* (Jacq.) J.F. Macbr. |
| **Pieridae** | *Abaeis nicippe* (Cramer, 1779) |  |  | **X** |  |  | *Russelia sarmentosa* Jacq. |
|  | *Anteos clorinde* (Godart, [1824]) | **X** |  |  |  |  | *Tecoma stans* (L.) Juss. ex Kunth |
|  | *Anteos maerula* (Fabricius, 1775) | **X** | **X** | **X** |  |  | *Russelia sarmentosa* Jacq. |
|  | *Ascia monuste monuste* (Linnaeus, 1764) | **X** | **X** | **X** |  | **X** | *Lantana camara* L. |
|  | *Eurema albula celata* (R. Felder, 1869) |  |  |  | **X** |  | *Cyanthillium cinereum* (L.) H.Rob. |
|  | *Eurema daira eugenia* (Wallengren, 1860) | **X** | **X** | **X** |  |  | *Bidens pilosa* L. |
|  | *Ganyra phaloe tiburtia* (Fruhstorfer, 1907) |  | **X** |  |  |  | *Dalbergia brownei* (Jacq.) Schinz |
|  | *Glutophrissa drusilla tenuis* (Lamas, 1981) |  | **X** |  |  |  | *Dalbergia brownei* (Jacq.) Schinz |
|  | *Nathalis iole iole* Boisduval, 1836 | **X** | **X** |  |  |  | Bidens pilosa L.; *Randia aculeata* var. *dasyclada* Steyerm. |
|  | *Phoebis agarithe agarithe* (Boisduval, 1836) |  |  | **X** |  |  | *Lysiloma divaricatum* (Jacq.) J.F. Macbr. |
|  | *Phoebis argante* ssp. n. DHJ01 (P. argante of Brown, 1929) |  |  | **X** |  |  | *Russelia sarmentosa* Jacq. |
|  | *Phoebis sennae marcellina* (Cramer, 1777) | **X** | **X** | **X** |  |  | *Russelia sarmentosa* Jacq. |
|  | *Pyrisitia nise nelphe* (Cramer, 1775) |  |  | **X** |  |  | *Russelia sarmentosa* Jacq. |
|  | *Pyrisitia proterpia* (Fabricius,1775) | **X** | **X** | **X** |  |  | *Tamonea curassavica* (L.) Pers. |
| **Riodinidae** | *Anteros carasius carasius* Westwood, 1851 |  |  |  | **X** |  | *Bidens pilosa* L. |
|  | *Calephelis perditalis donahuei* McAlpine, 1971 |  | **X** |  |  |  | *Bidens pilosa* L.; *Phyla nodiflora* (L.) Greene |
|  | *Calicosama lilina* (Butler, 1870) |  |  |  | **X** |  | *Sagittaria lancifolia* L. (M) |
|  | *Caria ino melicerta* Schaus, 1890 |  | **X** |  |  |  | *Caesalpinia bonduc* (L.) Roxb. |
|  | *Emesis tenedia* C. Felder & R. Felder, 1861 |  |  |  | **X** |  | *Cordia spinescens* L. |
|  | *Rhetus arcius thia* (Morisse, 1838) |  |  |  | **X** |  | *Cordia spinescens* L. |
|  | *Theope eupoli*s Schaus, 1890 |  |  |  | **X** |  | *Cordia spinescens* L. |
| **Sphingidae** | *Aellopos titan* (Cramer, 1777) | **X** | **X** | **X** | **X** |  | *Randia aculeata* var. *dasyclada* Steyerm. |
