# Supplementary figures and images for: Flower-mediated plant-butterfly interactions in an heterogeneous tropical coastal ecosystem
Source: PeerJ. 2018 Sep 7;6:e5493. doi: 10.7717/peerj.5493 (PMC6130237; doi:10.7717/peerj.5493)

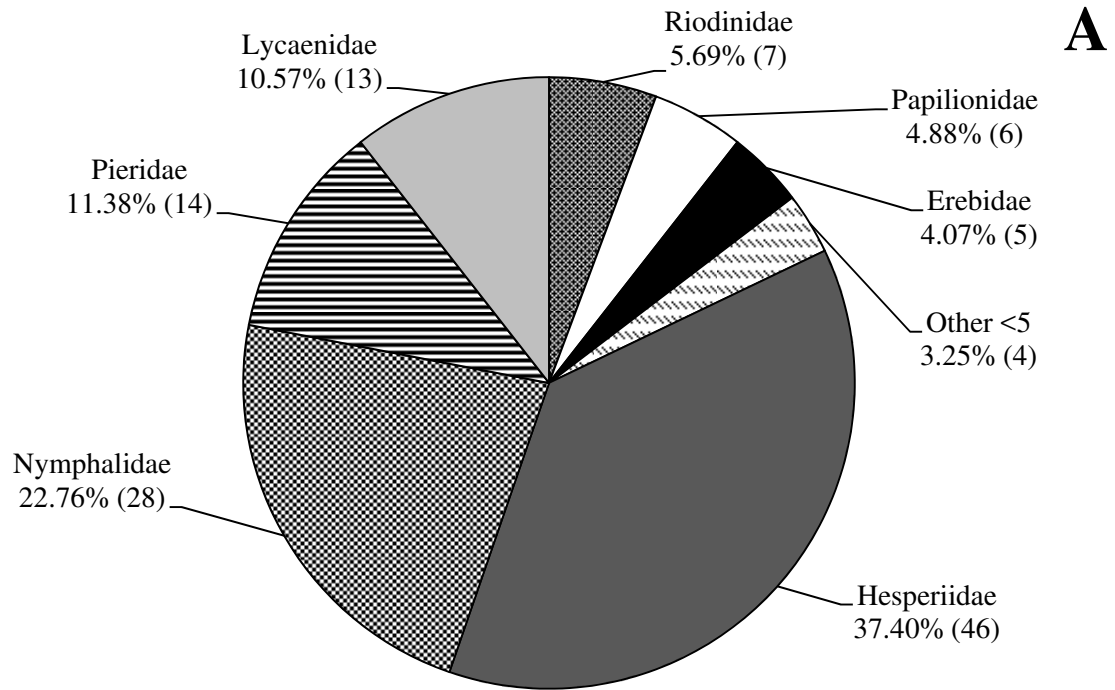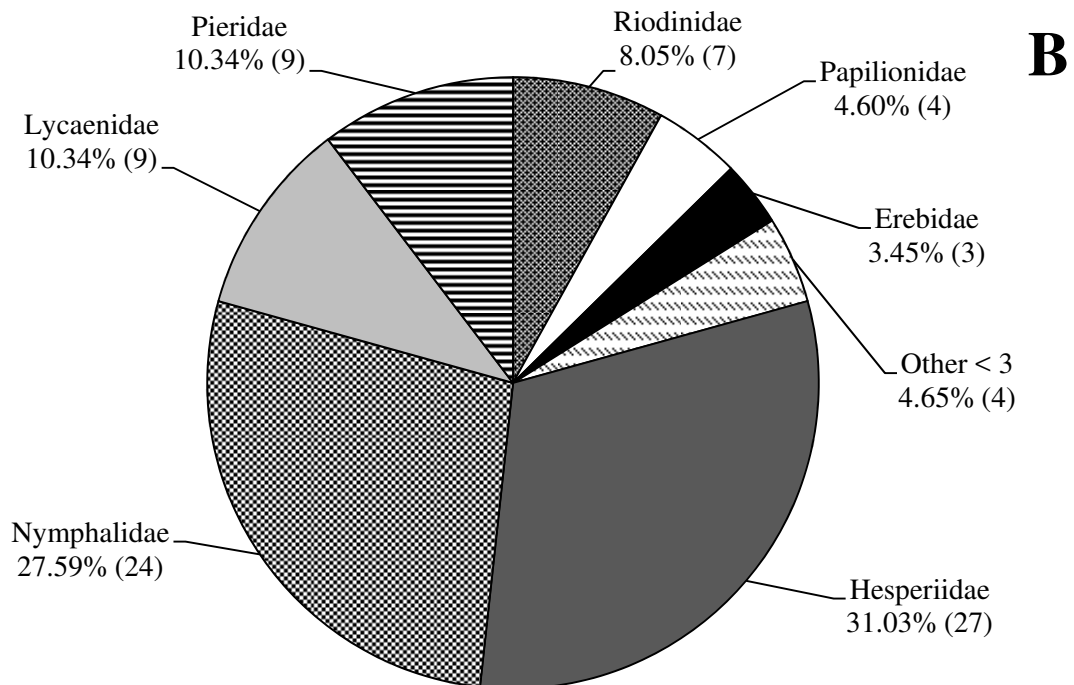

Supplement: Figure S1 — (A) Number of species per family, Nymphalidae, Hesperiidae, Pieridae, and Lycaenidae comprised 82.11% of all species, while the remaining 17.89% comprised four families with seven (or less) species per family. (B) Number of genera per family. We observed the same trend with Nymphalidae, Hesperiidae, Pieridae and Lycaenidae for number of genera, since these four families comprised 79.31%. The numbers of species and genera is shown in parentheses. [file peerj-06-5493-s002.pdf]
